# Factors associated with glycemic control among South African adult residents of Mkhondo municipality living with diabetes mellitus

**DOI:** 10.1097/MD.0000000000023467

**Published:** 2020-11-25

**Authors:** Charity Masilela, Brendon Pearce, Joven Jebio Ongole, Oladele Vincent Adeniyi, Mongi Benjeddou

**Affiliations:** a Department of Biotechnology, University of the Western Cape, Bellville; b Department of Family Medicine, Center for Teaching and Learning, Piet Retief Hospital, Mkhondo; c Department of Family Medicine, Walter Sisulu University, East London, South Africa.

**Keywords:** diabetes mellitus, glycemic control, Mkhondo municipality, South Africa, uncontrolled diabetes mellitus

## Abstract

This study examines the rate and the influencing factors of glycemic control among adult residents living with DM in Mkhondo Municipality of South Africa.

In this cross-sectional study, 157 individuals attending care for DM were recruited. Glycemic control status was categorized as poor if glycated hemoglobin (HbA1c) > 7% and very poor if HbA1c ≥ 9%. Multivariate regression analysis was used to identify the significant determinants of poor and very poor glycemic control.

The majority of the study participants were females (84.71%) and above 45 years old (88.55%). The overall prevalence of poor glycemic control was 77.71% (n = 122), while very poor glycemic control occurred in 50.6% (n = 80) of the study cohort. In the multivariate logistic regression model analysis, African traditional [AOR = 0.15; 95% confidence interval (95% CI) 0.04–0.57], fast food consumption (AOR = 5.89; 95% CI 2.09–16.81), elevated total cholesterol (TC) [odds ratio (OR) = 2.33; 95% CI 1.50–5.17], elevated low-density lipoprotein cholesterol (LDL-C) (AOR = 5.28; 95% CI 1.89–14.69), and triglyceride (TG) (AOR = 4.39; 95% CI 1.48–13.00) were the independent and significant determinants of poor glycemic control. Age (AOR = 0.46; 95% CI 0.23–0.92) was the only independent and significant determinant of very poor glycemic control.

We found a high rate of poor glycemic control (77.71%) possibly attributed to religious affiliation, fast food consumption, and dyslipidemia. On the contrary, about half of the study sample had very poor glycemic control (HbA1c ≥9%), which was predominant among younger cohort with diabetes mellitus. Interventions aimed at improving glycemic control in this population must also target religious practice, dietary patterns and dyslipidemia as well as tailored-approach for young people.

## 1 Introduction

The prevalence of diabetes mellitus (DM) in sub-Saharan Africa is increasing at an alarming rate and South Africa is at the forefront of the epidemic.^[1]^ Currently, the overall prevalence of type 2 diabetes in South Africa is estimated at 12.8%, which differs by geographical settings.^[2]^ Although some studies reported high prevalence of 26.6% to 60% among adult residents in urban settings,^[3–5]^ others reported figures as low as 7.6% in the rural and semi-rural areas of the country.^[6]^ Diabetes has a major impact on the lives of individuals, families, and public health.^[7]^ As such, South Africa has committed to lowering the burden of the disease in line with the National Development Plan.^[8]^ However, the magnitude and the speed in which diabetes has evolved in this country calls for emergency intervention.

Diabetes is defined as a complex metabolic disease that is characterized by chronic hyperglycemia.^[9,10]^ Although the pathophysiology of type 2 diabetes is not completely understood, impaired insulin secretion and increased insulin resistance, which may be a result of an interplay between environmental and genetic factors jointly, contribute to the development and progression of the disease.^[11,12]^ Over time, chronic hyperglycemia may lead to long-term damage and failure of various organs, progressive development of specific complications such as retinopathy, nephropathy, stroke, and cardiovascular diseases.^[13,14]^

Given the complex etiology of type 2 diabetes, its treatment and management require a multipronged approach that enables patients to achieve and maintain near normal glycated hemoglobin (HbA1c) levels.^[15]^ Studies have shown that achieving and maintaining normal levels of HbA1c is crucial in the prevention of microvascular complications, cardiovascular events, and associated morbidity and mortality.^[15,16]^ Compelling evidence suggests that achieving and maintaining the recommended glycemic levels requires the use of both oral and injectable anti-diabetic therapy.^[15]^ Furthermore, it has been suggested that combination therapy, with metformin and insulin, notably improves glucose control, lowers the incidence of cardiovascular diseases, and minimizes insulin requirements among patients with DM.^[17]^ When initiated early, combination therapy has demonstrated a long-term durability in comparison to any form of monotherapy.^[18]^ However, poor adherence to medications due to side effects, complications, frequent dosing, polypharmacy, and lack of education on diabetes self-management presents a great challenge in the management of DM.^[19,20]^ In addition, a negligent health care system that fails to intensify therapy appropriately when treatment goals have not been met may be a major contributor to poor glycemic control among patients.^[21]^

Diabetes is typically a life-long disease with incidence of death increasing steeply with duration of the disease.^[21]^ Also, patients who have been living with diabetes for a longer duration demonstrate an earlier onset of diabetes-related complications and tend to require intense pharmacological and nonpharmacological interventions.^[22]^ Therefore, it is unclear if the glycemic control status of individuals with DM differs by the duration of diagnosis and types of treatment modalities, especially among adult residents of rural communities of South Africa. More so, the influencing factors of glycemic control in individuals with DM in the rural Mkhondo municipality are poorly understood. These findings are needed to guide the crafting of context-specific interventions toward improving the clinical outcomes of people with DM in the region. The present study bridges the missing gaps by describing the sociodemographic and clinical profiles of individuals with DM, determines the rate and influencing factors of glycemic control among adult residents of Mkhondo Municipality in South Africa.

## 2 Methods

This cross-sectional study was conducted across three primary health care centers in the rural Mkhondo Municipality of Mpumalanga Province, South Africa. Mkhondo is a small resource-constrained border town situated between the Kingdom of Eswatini and the KwaZulu-Natal province of South Africa. The municipality is made up of 3 township and 3 government health facilities serving a combined population of 189,036 residents.

A total of 157 individuals attending chronic care for DM were recruited consecutively between January 2019 to June 2019. A sample size of 157 was estimated by using the formula for cross sectional study: {N = (Z1 – α)2 x P (1 - P) / D2}. Participants were eligible if they were at least 18 years old, had been on treatment for DM for a year, and had been attending regular follow-up visits at any of the 3 study sites. Pregnant and clinically unstable patients were excluded from the study.

Participants underwent face-to-face interviews using a standardized questionnaire, which comprised 3 major items, namely, demographic, lifestyle behaviors, and clinical data. The interviews were conducted by a trained research nurse who also performed anthropometric measurements (weight and height) according to standard protocols. The body mass index (BMI) of each participant was estimated and categorized as obese if BMI ≥30.0 kg/m^2^ or not. Clinical data were extracted from the medical records of each participant. In addition, fasting venous blood samples for lipid assays and glycated hemoglobin were drawn by the research nurse. All blood assays [HbA1c, total cholesterol (TC), low-density lipoprotein (LDL-C), triglyceride (TG), and high-density lipoprotein HDL-C)] were conducted by the National Health Laboratory Services (NHLS) in accordance with standardized protocols.

Poor glycemic control was defined as glycated hemoglobin (HbA1c) >7% in accordance with the guidelines of the Society for Endocrinology, Metabolism and Diabetes of South Africa (SEMDSA, 2017). In addition, participants with HbA1c ≥9% were further categorized as having very poor glycemic control.

Complete data for 157 participants were captured and analyzed by using the Statistical Package for Social Science (SPSS) version 25 for Windows (SPSS Inc., Chicago, IL). The sociodemographic and clinical characteristics of the participants were expressed as mean ± standard deviation for continuous variables and frequency (percentages) for categorical variables. The associations between the demographic, lifestyle behaviors, and glycemic control were examined at different cut-offs; first at HbA1c >7% (poor glycemic control) versus good glycemic control (HbA1c ≤7%), followed by HbA1c ≥9% (very poor glycemic control) versus fair glycemic control (HbA1c <9%) by using a Chi-square test. Multivariate odd ratios (crude and adjusted), using logistic regression model analysis, were estimated with their 95% confidence intervals (95% CIs) to identify the independent and significant determinants of poor and very poor glycemic control. A *P* value < .05 was considered for statistical significance.

## 3 Results

The majority of the participants were female (84.71%), above 45 years old (88.55%), Zulu-speaking (82.80%), practicing Christians (87.26%), employed (68.78%), had attained post-primary education (75.16%), never smoked cigarettes (86.62%) nor consumed alcohol drink (77.07%), consumed fruits and vegetables weekly (98.09%), consumed fast food weekly (67.52%), and engaged in a sedentary lifestyle (65.61%) (Table 1).

**Table 1:** Demographic characteristics of the study participants.

Variables	Frequency (n)	Percentage (%)
Sex		
Male	24	15.29
Female	133	84.71
Age, yr		
18–25	03	1.91
26–35	08	5.09
36–45	07	4.45
46–55	40	25.47
56–65	52	33.12
≥66	37	23.56
Ethnicity		
Zulu	130	82.80
Swati	27	17.20
Religion		
Christianity	137	87.26
African Traditiona1	20	12.74
Employment status		
Employed	108	68.78
Unemployed	49	31.21
Educational level		
Primary	39	24.84
Post primary	118	75.16
Smoking status		
Never smoked	136	86.62
Ever smoked	21	13.38
Alcohol consumption		
Never drank	121	77.07
Occasional	36	22.93
Fruit and vegetable Consumption		
1–3 times/week	154	98.09
Never	03	1.91
Fast food consumption		
Never	51	32.48
1–3 times/week	106	67.52
Physical activity		
Active	54	34.39
Inactive	103	65.61

**Figure 1:**
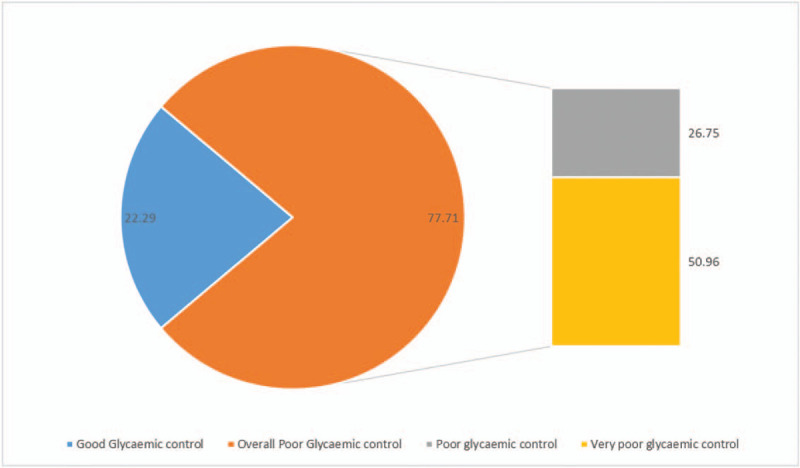
Overall poor glycemic control status of the participants.

Overall, the majority of the participants had poor glycemic control (77.71%) and about half of the participants (n = 80) had very poor glycemic control (Fig. 1). The rate of poor and very poor glycemic control differed by sociodemographic and clinical characteristics (Table 2). In Chi-square analysis, there was a significantly higher risk of poor glycemic control in individuals who were Christians, consumed fast food, had elevated TC, elevated LDL-C, and elevated TG. Beside age, all other participants’ characteristics were not significantly associated with the risk of having very poor glycemic control.

**Table 2:** Chi-square test showing associations between glycemic control and sociodemographic and clinical factors.

	Good glycemic control	Poor glycemic control	*P*	Fair glycemic control	Very poor glycemic control	*P*
Variables	≤7% HbA1C	>7 HbA1c		<9 HbA1c	≥9 HbA1c	
Gender			.472			.586
Male	04 (16.67)	20 (88.33)		13 (54.17)	11 (45.83)	
Female	31 (23.31)	102 (76.69)		64 (48.12)	69 (51.88)	
Age, yr			.080			.026
<55	10 (15.38)	55 (84.62)		25 (38.46)	40 (61.54)	
≥55	25 (27.17)	67 (72.83)		53 (56.99)	40 (43.01)	
Employment status			.426			.748
Employed	26 (24.07)	82 (75.93)		53 (44.17)	67 (55.83)	
Unemployed	09 (18.37)	40 (81.63)		24 (64.86)	13 (35.14)	
Educational Level			.142			.991
Primary	12 (30.77)	27 (69.23)		22 (56.41)	17 (43.59)	
Post Primary	23 (19.49)	95 (80.51)		55 (46.61)	63 (53.39)	
Religion			.001			.289
Christianity	25 (18.25)	112 (81.75)		63 (46.32)	73 (53.68)	
African Traditional	10 (50.00)	10 (50.00)		17 (70.83)	07 (29.17)	
Fruit and vegetable consumption			.643			.529
1–3 times/week	34 (22.08)	120 (77.92)		75 (48.70)	79 (51.30)	
Never	01 (33.33)	02 (66.67)		02 (66.67)	01 (33.33)	
Fast food consumption			.007			.538
Never	18 (35.29)	33 (64.71)		30 (58.82)	21 (41.18)	
1–3 times/week	17 (16.04)	89 (83.96)		47 (44.34)	59 (55.66)	
Physical activity			.775			.089
Active	15 (23.43)	49 (76.56)		27 (42.19)	37 (57.81)	
Inactive	20 (21.50)	73 (78.49)		50 (53.76)	43 (46.24)	
Total Cholesterol			.035			.135
<4.5 mmol/L	24 (28.92)	59 (71.08)		44 (53.01)	39 (46.99)	
≥4.5 mmo/L	11 (14.86)	63 (85.14)		33 (44.59)	41 (55.41)	
HDL-C			.356			0.292
≥1.2 mmol/L	17 (19.54)	70 (80.46)		38 (43.68)	49 (56.32)	
<1.2 mmol/L	18 (25.71)	52 (74.29)		39 (55.71)	31 (44.29)	
LDL-C			.000			.134
<1.8 mmol/L	27 (37.50)	45 (62.50)		40 (55.56)	32 (44.44)	
≥1.8 mmol/L	08 (9.41)	77 (90.59)		37 (43.53)	48 (56.47)	
Triglycerides			.000			.133
<1.7 mmol/L	20 (40.00)	30 (60.00)		28 (56.00)	22 (44.00)	
≥1.7 mmol/L	15 (13.64)	95 (86.36)		48 (45.28)	58 (54.72)	
Duration of diagnosis			.573			.068
< 5 yr	28 (23.33)	92 (76.67)		61 (50.83)	59 (49.17)	
≥5 yr	07 (18.92)	30 (81.08)		16 (43.24)	21 (56.76)	
Treatment Regime			.126			.419
Oral	34 (29.94)	108 (76.06)		72 (50.70)	70 (49.30)	
Insulin/ Insulin + Oral	01 (6.67)	14 (93.33)		05 (33.33)	10 (66.67)	
Hypertension			.379			.201
No	07 (29.17)	17 (70.83)		14 (58.33)	10 (41.67)	
Yes	28 (21.05)	105 (78.95)		63 (47.37)	70 (52.63)	
Obesity			.809			.323
No	11 (21.15)	41 (78.85)		23 (44.23)	29 (55.77)	
Yes	24 (22.86)	81 (77.14)		54 (51.43)	51 (48.57)	

In the multivariate (crude) logistic regression model analysis (Table 3), African traditional religion, consumption of fast food, elevated TC, elevated LDL-C, and elevated TG were the independent and significant determinants of poor glycemic control. Similarly, after adjusting for other covariates (Table 3), the magnitude and direction of association remained for African traditional religion, consumption of fast food, and LDL-C; however, TC became insignificant, while the direction of association changed for TG. Patients who were practicing African traditional religion were less likely to have poor glycemic control compared with those practicing Christianity. However, patients with elevated LDL-C were 5 times more likely to have poor glycemic control than those with normal LDL-C. Similarly, patients with elevated TG were 4 times more likely to have poor glycemic control than those with normal TG.

**Table 3:** Adjusted and unadjusted logistic regression models showing sociodemographic and clinical factors associated with poor glycemic control glycemic control (HbA1C>7%).

Variables	Unadjusted odds ratios (95% CI)	Adjusted odds ratios (95% CI)
All		
Gender		
Male	1	1
Female	1.50 (0.48–4.78)	2.12 (0.51–8.89)
Age, yr		
<55	1	1
≥55	0.48 (0.22–1.10)	0.75 (0.27–2.13)
Ethnicity		
Zulu	1	1
Swati	1.32 (0.46–3.78)	1.30 (0.31–5.39)
Employment status		
Employed	1	1
Unemployed	1.40 (0.60–3.28)	1.07 (0.35–3.16)
Religion		
Christianity	1	1
African traditional	0.22 (0.84–0.59)^∗^	0.15 (0.04–0.57)^∗^
Fast food consumption		
Never	1	1
1–3 times/week	2.85 (1.31–6.19)^∗^	5.89 (2.09–16.81)^∗^
Total cholesterol		
<4.5 mmol/L	1	1
≥4.5 mmol/L	2.33 (1.50–5.17)^∗^	1.24 (0.39–3.23)
LDL-C		
<1.8 mmol/L	1	1
≥1.8 mmol/L	5.77 (2.41–13.79)^ †^	5.28 (1.89–14.68)^∗^
Triglycerides		
<1.7 mmol/L	1	1
≥1.7 mmol/L	0.25 (2.41–13.79) ^†^	4.39 (1.48–13.00)^∗^
Duration of diagnosis		
<5 yr	1	1
≥5 yr	1.30 (0.57–3.29)	1.05 (0.34–3.19)
Treatment regime		
Oral	1	1
Insulin/Oral + Insulin	4.40 (0.55–34.75)	0.60 (0.59–7.00)

In the multivariate (crude and adjusted) logistic regression model analysis (Table 4), very poor glycemic control (HbA1c ≥ 9%) was compared with fair glycemic control (HbA1c < 9%), it was also found that only the age of the participants was significantly associated with the risk of having very poor glycemic control. Older patients (≥55 years) were less likely to have very poor glycemic control in comparison to the younger individuals.

**Table 4:** Adjusted and unadjusted logistic regression models showing sociodemographic and clinical factors associated with very poor glycemic control (HbA1C ≥9%).

Variables	Unadjusted odds ratios (95% CI)	Adjusted odds ratios (95% CI)
All		
Gender		
Male	1	1
Female	0.78 (0.32–1.87)	0.70 (0.27–1.78)
Age, yr		
<55	1	1
≥55	0.48 (0.25–0.91)^∗^	0.46 (0.23–0.92)^∗^
Ethnicity		
Zulu	1	1
Swati	0.87 (0.38–2.00)	0.73 (0.28–1.89)
Employment status		
Employed	1	1
Unemployed	1.0 (0.51–1.97)	0.76 (0.36–1.58)
Duration of diagnosis		
<5 yr	1	1
≥5 yr	1.35 (0.64–2.85)	1.28 (0.59–2.27)
Treatment regime		
Oral	1	1
Insulin/Oral + Insulin	2.05 (0.66–6.32)	2.33 (0.66–8.24)

## 4 Discussion

In the current study, we examined factors influencing glycemic control in individuals with DM in the rural Mkhondo municipality, South Africa. This is largely a rural, resource-constrained setting and an understudied region of the country. The overall prevalence of poor glycemic control was 77.71%. This finding is worse than previous reports from South Africa^[3–6]^ where the prevalence of poor glycemic controlled ranged from 7.6% to 60.0%; however, it is better than the 82.35%^[23]^ and 83.8%^[24]^ reported in the rural and semi-urban communities in the Eastern Cape, South Africa. In comparison to other studies conducted in other African countries, the rate reported in this study is better than previous reports from Ghana (86.4%)^[25]^ and Sudan (85.0%).^[26]^ Unfortunately, it is worse than rates reported in Ethiopia (70.8%)^[27]^ and Kenya (60.5%).^[28]^ The high prevalence of poor glycemic control may be due to low awareness of the disease as well as suboptimal treatment that is often observed among rural dwelling populations.^[23–26]^ Our findings highlight the need to intensify glycemic control in individuals with DM, given the life-threatening and economic impacts of the disease.

There is an overwhelming amount of evidence with regards to how religion and spirituality influence glycemic control across different populations.^[27,29]^ Some studies suggest that most spiritual individuals who are living with DM present poor self-care in comparison to their nonspiritual counterparts.^[27]^ However, the extent to which the African traditional religion influences glycemic control among South Africans is poorly understood. Our findings suggest that affiliation with the African traditional religion is associated with poor glycemic control (HbA1c >7%). Although our study highlights the need to consider and address religious practices of patients in the care for DM, further studies are recommended so as to gain a better insight into the effect of religious practices on glycemic control.

Medical nutrition therapy is an important aspect of diabetes management. As such, diets rich in sugar, refined carbohydrates, and high in fat have been associated with the incidence of diabetes.^[30,31]^ In the current study, weekly consumption of fast food was associated with poor glycemic response. These findings corroborate previous observations made in the United Arab Emirates, where fast food consumption was an independent predictor of poor glycemic control in patients with DM.^[32]^ The study further demonstrated that consuming fresh fruits could have a protective effect on glycemic control among patients. Although fruit and vegetables consumption were investigated in the present study, no association was established with poor glycemic control. Future studies with a larger cohort of patients with DM might provide more insight into the association between glycemic status and consumption of fruits and vegetables.

Diabetic dyslipidemia, characterized by high plasma triglycerides, high LDL-cholesterol, and low HDL-cholesterol, is associated with poor glycemic control and cardiovascular risk.^[33]^ As such, tight glycemic control in patients with DM may lead to an improved lipid profile and a reduction in cardiovascular disease risk.^[33,34]^ It was also demonstrated that aggressive therapy, which includes statin and lifestyle interventions aimed primarily at lowering LDL-C, do improve glycemic control among patients with DM.^[35]^ In the current study, increased TC, LDL-C, and triglycerides were associated with poor glycemic control. However, we do not know whether the study population was initiated on statin therapy. As a result, the lipid-lowering effect of statins was not evaluated.

Insulin resistance and beta cell dysfunction are the most prominent metabolic features of type 2 diabetes.^[9]^ Moreover, early initiation of insulin, alone or in combination with metformin, has been shown to improve glycemic control and preserve pancreatic β-beta cell function in patients with DM.^[15,17]^ In the present study, no significant effect on glycemic control was observed among patients receiving insulin alone or in combination with metformin. The lack of association observed may be a result of both the patient and clinician's reluctance to initiate insulin therapy because of perceived safety issues such as weight gain and hypoglycemia.^[36]^ Also, poor compliance with treatment by patients and lack of potency of insulin as a result of improper storage may have contributed to the lack of effect observed.

In the current study, age (<55 years) was associated with very poor glycemic control (HbA1c ≥9%). It is plausible that younger patients may not yet internalize the chronicity of the disease and by implication, adjust to the necessary lifestyle changes including compliance with clinic visits and adherence to medications. DM is considered a disease of the elderly; however, studies conducted in China^[37]^ and the United States of America showed that suboptimal glycemic control was more common in younger adults,^[35]^ due to poor adherence to medication and clinic appointments.^[38]^ Hence, our finding is consistent with the previous reports. Given the unique characteristics of DM among older adults, previous studies have shown that achieving good glycemic control in older adults requires a tailored therapeutic approach that will eliminate the risk of cardiovascular disease and hypoglycemia.

## 5 Limitations

Given the small sample size and the cross-sectional design of the study, the identified determinants should not be considered as causation. Notwithstanding, the small sample included, this study was conducted in 3 primary health care centers serving the predominant rural communities of Mkhondo Municipality. As such, the findings are generalizable to the population of individuals living with DM in the region and similar settings in the country. This is the first study to report the rate of glycemic control and its influencing factors in the rural Mkhondo Municipality of South Africa.

## 6 Conclusion

We found a high rate of poor glycemic control (77.71%) possibly attributed to religious affiliation, fast food consumption, and dyslipidemia. On the contrary, about half of the study sample had very poor glycemic control (HbA1c ≥9%), which was predominant among younger cohort with DM. Interventions aimed at improving glycemic control in this population must also target religious practice, dietary patterns, and dyslipidemia as well as tailored-approach for young people. These findings would guide the local authorities and clinicians in crafting and implementing appropriate interventions to improve the clinical outcomes in people with DM in the region.

## Acknowledgments

The authors would like to thank the study participants, Piet Retief Hospital, Thandukukhaya Community Health Center, Mkhondo Town Clinic, and the Department of Health of Mpumalanga.

## Author contributions

CM, BP, JJO, and MB conceptualized, designed, and implemented the study protocol. CM and OVA analyzed the data and drafted the manuscript. All authors revised and approved the final draft of the manuscript for submission.

**Conceptualization:** Charity Masilela, Brendon Pearce, Joven Jebio Ongole, Mongi Benjeddou.

**Formal analysis:** Charity Masilela, Oladele Vincent Adeniyi.

**Investigation:** Charity Masilela.

**Project administration:** Oladele Vincent Adeniyi.

**Resources:** Mongi Benjeddou.

**Supervision:** Mongi Benjeddou.

**Writing – original draft:** Charity Masilela.

**Writing – review & editing:** Oladele Vincent Adeniyi.
